# Women’s experiences of living with involuntary childlessness in Uganda: a qualitative phenomenological study

**DOI:** 10.1186/s12905-022-02087-0

**Published:** 2022-12-19

**Authors:** Susan Asiimwe, Charles Peter Osingada, Scovia N. Mbalinda, Mark Muyingo, Elizabeth Ayebare, Mariam Namutebi, Patience A. Muwanguzi

**Affiliations:** 1 Department of Nursing, School of Health Sciences, College of Health Sciences, Makerere University, Kampala, Uganda; 2 Department of Obstetrics and Gynecology, School of Medicine, College of Health Sciences, Makerere University, Kampala, Uganda

**Keywords:** Involuntary Childlessness, Women, s experiences

## Abstract

**Background:**

Involuntary childlessness is a global phenomenon that negatively impacts the couple, or the family involved. The experiences of women living with involuntary childlessness have not been well documented in the literature, specifically in the Ugandan context. The purpose of the study was to explore the experiences of women living with involuntary childlessness in Uganda.

**Methods:**

A qualitative phenomenological approach was used. Fifteen in-depth interviews were conducted among women experiencing involuntary childlessness attending a National Referral Hospital. Purposive sampling was, and data saturation determined the actual sample size. Thematic analysis was used for data analysis. The results are presented in the form of text and narrative quotes from participants.

**Results:**

Six themes emerged (i) Inadequate social support (ii) psychological torture (iii) continued grief (iv) marital instability (v) failure attributed to childlessness and (vi) financial constraints. Inadequate social support was in the form of having an unsupportive partner, altered social relation, and altered social status, while women experienced name-calling, emotional abuse, stigma, and blame under the psychological torture theme. Women experienced feelings of distress and grief, including anger, irritability, sadness, stress, and feelings of despair. Women with involuntary childlessness recounted experiencing unstable marriages characterized by infidelity, divorce, abandonment, and polygamous marriages. Some women coped positively, while others employed negative coping strategies such as social withdrawal and isolation. Women who their partners and families well supported coped positively. In contrast, those who did not receive as much support were stressed, sad, angry, and had lost hope of pregnancy.

**Conclusions:**

In this study, women with involuntary childlessness lacked social support amidst experiences of marital turmoil, psychological torture, feelings of distress and grief, unfulfilled motherhood expectations, and financial constraints while seeking treatment, therefore, there is a need to screen the women for psychological / mental illness symptoms and provide empathetic care and counseling. The prevalence of involuntary childlessness is not well documented in Uganda and a study can be done to determine its extent.

## Background

Involuntary childlessness is defined as the inability to conceive after 12 months of unprotected sex or a woman’s inability to carry a pregnancy to term [1]. It is a global phenomenon that has a negative impact on the couple, or the family involved [2]. Women are particularly affected by the consequences of involuntary childlessness, including intimate partner violence, mental illness, and lack of social support [3].

Globally, the estimated number of couples with involuntary childlessness increased from 42.0 million couples to 48.5 million couples in 2010 [4]. In addition, 19.2 million couples were reported to have primary infertility. The global prevalence of infertility ranges from 8 to 12% [5, 6]. In Africa, the burden of childlessness is increasing, with an estimated prevalence of about 30–40% [6, 7]. In Uganda, about 10–15% of couples experience involuntary childlessness [8]. This translates to an estimated five million couples with infertility in Uganda. In addition, involuntary childlessness is one of the leading indications for gynaecologic clinic visits in Uganda [9].

In Uganda, the existing pronatalist ontology dictates that the sole purpose of marriage is for procreation[10]. Children in an African context provide social security in old age, stability in marriage, support, domestic labor, wealth, family continuity, social status, and profound identity of motherhood[11]. Consequently, the blame and stigma often fall on the woman for failure to provide children to the family, even in cases when the involuntary childlessness is due to male factors[12]. Studies from other settings cite that women with involuntary childlessness may experience feelings of grief, envy, depression, anxiety, reduced life satisfaction, identity and inadequacy concerns, and lack of personal control, among others[12, 13]. A limited number of studies in Uganda have explored the experiences of women with involuntary childlessness. The importance of social-cultural settings, especially in involuntary childlessness in shaping values attached to children, reproductive technology, and family support system calls for the need to provide context-specific experiences of women with involuntary childlessness in Uganda.

The study adds to the body of knowledge vital in providing rich-description and context-specific understanding of women's experiences with involuntary childlessness. The findings from this study may empower healthcare providers to effectively respond, develop effective interventions, and improve women's experiences with involuntary childlessness. Therefore, the purpose of this study was to explore the experiences and coping strategies of women living with involuntary childlessness attending a fertility clinic in Uganda.

## Methods

### Study design

This was a qualitative phenomenological study that was conducted to explore the lived experiences and coping strategies of women with involuntary childlessness.

### Study setting

The study was conducted at Kawempe National Referral hospital. Kawempe National Referral Hospital is in Kawempe Division, one of the five administrative units of Kampala Capital City Authority. The hospital offers specialized obstetrics and gynecology services in Uganda [14]. Kawempe National referral hospital is the biggest hospital for obstetrics and gynecology with over 22,000 births annually. It also offers care to over 3600 women with gynecological issues each year. Consultations for infertility related problems account for 55% of all the gynecological consultations. The hospital employs gynecologists who offer services related to infertility such as investigations for the cause, and treatment. However, artificial reproductive technologies are not provided at the hospital. In Uganda, generally, this service can be accessed only in private facilities where women pay around 5000 USD or more for IVF and other treatments.

### Study population

The study was conducted among women who were seeking fertility treatment at Kawempe National Referral Hospital. These included women who had lived with involuntary childlessness for a period of more than one year and those with no living child. Participants were sampled until data saturation was reached, when no new information was collected. In this study, data saturation was reached after 15 participants were interviewed. We used purposive sampling to obtain a rich description of experiences of women with involuntary childlessness. The criteria for purposive sampling were based on the number of years lived with involuntary childlessness.

### Data collection

Data were collected using in-depth interviews. A total of 18 participants were approached, and only 15 were accepted to participate in the study. *The 2 exclusion were on the basis of the number of years they had lived with involuntary childlessness, while 1 it was on the basis of language participant was neither able to speak English nor Luganda the local language.* Participants were interviewed in Luganda, a widely spoken local dialect in the study area. Participants were identified and sampled from the gynecologic clinic register. Upon obtaining consent, participants were interviewed using an interview guide. Additional support was sought from the participants to audio-record the interviews. Socio-demographic information was collected from the participants using a semi-structured questionnaire before starting the in-depth interview. The interview guide included questions that explored and probed for women's experiences and coping strategies with involuntary childlessness. The first author conducted the interview, a midwife by training, and a standby counsellor was also present for the participants who may have emotional outbursts during the interview. In cases of emotional outbursts, the interview was paused or postponed to a later time. This was done to allow for recovery from the emotional turmoil.

### Data management and analysis

The audio-recording was transcribed verbatim and translated from Luganda to English. Data was manually analyzed using the six steps of thematic analysis from seminal work by Braun and Clarke. ref Firstly, the first author read the transcript several times to gain a deeper understanding of the data. Subsequently, codes and emerging recurrent themes were identified from the data. Data analysis was done by two authors: S.A and PAM, which ensured confirmability with the interpretation of the data.

## Results

Table 1 presents the characteristics of study participants.

**Table 1:** Social demographics characteristics

Participant’s characteristics	Frequency	Percentage (%)
Age		
20–24	2	13.3
25–29	3	20
30–34	3	20
35–40	7	46.6
Duration of involuntary childlessness (years)		
2–4 years	3	20
5–7 years	6	40
8–10 years	2	13.3
11–15 years	3	20
16 years and above	1	6.6
Marital status		
Married	14	93.3
Separated/ divorced	1	6.7
Occupation		
Self-employed	12	80
Housewife	3	20
Level of education		
No Education at all	1	6.7
Primary	1	6.7
Secondary	10	66.6
Tertiary	3	20
Religion		
Muslim	6	40
Catholic	3	20
Protestant	3	20
Born again	3	20

### Experiences of women with involuntary childlessness

Six major themes were identified from in-depth interviews: inadequate social support, marital instability, psychological torture, continued grief, failure attributed to childlessness, and financial constraints (Table 2).

**Table 2:** Coding tree for the experiences of women living with involuntary childlessness

Themes	Categories	Codes
Inadequate social support	Perceived change in social status	I am not allowed to use our property like land
		I am not considered as a wife
		They do not involve me in making decisions that concern our family
		I am not ever invited for family meetings and gathering
		My In-laws sent me away from my marital home
		I have pressure from in-law on the husband’s side to give them a child
		My in-laws and friends are getting women for my husband
	Altered Social relations	I cannot attend social gatherings, including weddings, birthday parties
		My husband cannot take me to social gatherings
		All of my in-laws do not visit our home
		My friends are hiding from me
		I do not have genuine friends
		They do not involve me in topics related to children and other community involvement
		Everyone gave up on me
Marital instability	Infidelity	My marriage is considered temporary because I do not have a child
		My husband has requested to marry another woman
		My husband married another woman
		My husband doesn’t come back home
		My husband has extra-marital relations
	Lack of partner support	My husband refused to visit the hospital with me
		My partner doesn’t buy food; he refused to provide all the basic needs, he doesn’t give me money for treatment
		My husband is not patient
Psychological torture	Verbal abuse	My husband abused me that I am a witch
		People in my community call me terrible names, and so my in-laws
		Being called a thief, a person with bad luck, a barren woman, a prostitute, a witch
	Emotional Abuse	I am a topic of discussion in my neighborhood,
		No empathy from the doctors
		People see me as an enemy
		I am despised by my in-laws
		They see me as a problem
		I am blamed for not giving birth
	Internalized Stigma	I feel like a problem to
		I no longer want to see any of my friends
		They think I am an enemy
		They say I am wasting my husband’s money and food
Continued Grief	Feeling emotional	I feel stressed
		I have anger towards myself
		I am unhappy
		I feel sad all the time
		I cannot stop hurting
	Feelings of Hopelessness and loss	I have pain that I can’t express as they call me selfish
		I feel the pain of loss
		I feel terrible
		I feel rejected by my friends
		I have lost hope
		I feel like God has forsaken me
		I cry all the time
Failure attributed to childlessness	Failed expectations	I have not only failed myself but also failed the community expectations
		I have failed my partner and family expectations
	Unmet expectations of motherhood	I wish I had a baby
		I cannot control my mind, I think of a baby all the time
		My husband is always lamenting about not having a child
Financial constraints	Expensive medicines and investigations	I cannot afford the laboratory tests and other investigations
		I did not buy all the drugs that were prescribed
	Unaffordable treatments and procedures	I did not do for the procedure because I couldn’t afford it
		I keep postponing treatment
		I cannot afford to undergo IVF

### Inadequate social support

The following categories were identified; perceived change in social status and altered social relations.

#### Perceived change in social status

Some women were no longer considered as wives in their marital homes, and they were not part of the property that they attained with their husbands. Most women were not consulted about decisions concerning their home, and they were always left behind during meetings and gatherings by their partners.

Some participants stated that they are left behind during any functions and gatherings because of not having children. One participant stated,

He does not take me for any functions or holidays …. I stay here or go to spend it with my relatives … his attitude is like … “…if I am to take you home…taking you as who…? For him, it is like that being without a kid is like … I do not know how to express it … she continues, but still, also I avoid such ceremonies because my heart is very weak…I cry very easily …. (Participant 13)

In addition, other participants stated that they were not only excluded from social gatherings but also denied access and utilization of the property of their husbands.

…….. You find that when a man buys something … an asset … he registers it in his names because you do not have a child even when I have contributed my money….he has land, but he cannot even let me plant beans…. His mother is using it….hmmm…. (Participant 2)

Furthermore, other participants stated that their In-laws reached the extent of advising their husbands to remarry or get them women searching for a child. Most of them lived in polygamous marriages. In this regard, participant 3 narrated that:

When you have no child, you are not considered as a wife to their brother…. When my husband decided to wed me... my father in-law asked him why he is wedding me before we get children…up to now they have never taken me as a wife to their brother …. (Participant 3)

#### Altered social relations

Almost all the participants reported alterations in their social relations; women deliberately refused to attend functions or meet up with family for any social gathering. One participant narrated,

……you can approach a friend and she/he advises you try this or that ….. But as for my husband’s family … they cannot support you because it’s you who is with a problem, so you must fight your own battle. As you know men, for them they can get another woman….. (Participant 1)

Some participants were yearning for social support from friends, while others were disappointed by friends who give impractical advice. See the following excerpts.

….. I have friends but most of them discourage me, they tell you words you would not want to hear, like why don’t you leave that man maybe he is the one with a problem, just leave come and we hustle outside marriage, what have you gained in that marriage? and that is not what I want to hear… I want true friends; they tell everyone my problems and I even hate meeting people I know (participant 3).

Some other participants had excluded themselves from functions and other gatherings that remind them of their childlessness. Participant 6 expressed anger when they stated:

….. Children’s birthday parties? I do not go there, why should I go there?.... of course, I feel bad … why is it that I do not produce when I am a woman and meant to produce children… (Participant 6)

Other participants reported having been abandoned by their friends because of the inability to have children. For example, Participant 3 expressed her disappointment,

… It hurts…. people you call your close friends … classmates, you grew up together….. they all abandon you because you are infertile … let me narrate to you what happened …I had a very close friend who was also my matron at our wedding … when we were still friends she had a problem and we would meet and pray together … both of us were Christians … we would pray together … but when she got pregnant and gave birth to a baby. .. she abandoned me … when I call her, she does not pick and when I go to her home, I do not find her there…she no longer has time for me she is busy with her children…. (Participant 3)

### Marital instability

Most women lived in fear of losing their marriage to other women; some were not sure of the longevity of their current marriages.

#### Infidelity

Some women mentioned that their husbands are in intimate relationships outside their marriage, while other participants lived in polygamous marriages.

My husband no longer respects our marriage …. He moves out with different women and has several women he is dating… I read his messages and it was disappointing…and I can’t talk about it because they will call me selfish… but it hurts ….. (Participant 6)

#### Lack of partner support

Most participants reported that they were not supported well by their partners in terms of the provision of basic needs for a home. They considered this lack of support to be a result of the involuntary childlessness which they were experiencing. The following two excerpts speak to this challenge.

… time reached when my husband would just wake up and leave…..he could not buy food or anything I needed for home….my mother would send me food but time reached and I decided to go back to my home and I left him…. (Participant 3)

### Psychological torture

Many women mentioned having been tortured, abused emotionally and verbally. The abusive comments were from in-laws, their husbands, and the community at large.

#### Emotional abuse

Participants reported that lack of empathy from health workers makes this experience harder as doctors break bad news without any mental preparations. This is highlighted below,

I went to the hospital to seek treatment, but the doctor talked to me without any empathy… I felt so bad… he told me I will never give birth without any explanation of the results ….. I cried and went back home… but my friend told me about a doctor here who can solve my problem and it is the reason I came…. (Participant 3)

The respondents further revealed that the public had labeled them with terrible names. They have become a topic of discussion in their villages, and this makes the whole experience unbearable. One participant shared,

In my community, I do not talk to anyone….they think I am a witch because I am a barren woman... and I do not disclose my problems …. Because when you tell a friend they tell others and you become a topic of discussion…. I, therefore, keep in my house and I do not visit……(Participant 5)

#### Verbal abuse

Many women have been verbally abused by their immediate family members, extended family, and community. These verbal abuses contribute to psychological torture. For instance,

…. there are some words my husband told me which still hurt me up to now ….“since you have failed to give me a child, why don’t you bring your sister instead…”….’’ bring your sister so that we get a child…” not thinking that this suggestion hurts me…the thought of your sister sleeping with your husband to get a child…! (Participant 3)

#### Internalized stigma

Some participants felt stigmatized; women perceived that their communities and family see them as problems, perceived as enemies, and felt they were a waste of resources. Most women reported excluding themselves from social gatherings, including others where children were present. However, some women would be left behind by their husbands and other family members. Women felt they were a disgrace and humiliation to their families.

#### I avoid everyone because I think I am seen as a problem, even when I find them amidst a conversation they stop talking until you leave and you hear them laughing………

She continued; my in-laws tell me that I am a waste of time and money since I can’t give their brother a child….. Yet it’s me who even works hard to provide most things to my family….. (Participant 10)

### Continued grief

Most of the participants were frustrated during the interview. They reported continued feelings of stress, anger, sadness/ unhappiness, heart ache, pain that cannot be expressed, the pain of loss, feeling terrible, rejected, loss of hope, and hope feelings of God forsaking them.

#### Feelings of hopelessness and loss

Some participants described life to be hard and difficult, especially when the support is limited:

...that kind of life has not been easy…life has been difficult….. in that time comes when even the person who has been comforting you eventually also gives up………… there is a time when you see that there is no one on your side and even God has forsaken you when you need Him most…you are without any hope…you feel hopeless.(Participant 3)

Other participants reported pain that they could not express or describe. The pain of loss when their husbands moved out with other women or married other women searching for children. One participant described this:

…. My husband left me and married another woman… I felt it was fair, but the pain still comes …. You feel that much pain of losing him to another woman…I cannot even tell anyone of that pain because they will call me selfish …. So I think you can imagine how a woman who cannot bear a child feels….. (Participant 3)

### Failure attributed to childlessness

Some of the participants reported having failed to meet the expectations of womanhood as expected from their husbands, in-laws, and even to themselves. All the participants mentioned that the sole reason for getting married was to bear children but failed to.

Some of the participants reported having failed to meet the expectations of their husbands, in-laws, and even of themselves.

…. at home…like you know when you get married, it is natural for your in-laws to expect you to produce children and when things do not go like you expect you feel you are not doing what brought you into marriage…( Participant 5)

Most of the participants felt disappointed as they could not bear children within their marriage, making them feel like they have not met their obligation of having been married.

….. I left my parents with their blessing to get married to my husband… and automatically I knew the next thing would be starting a family and having children. This did not happen as I had envisaged… I have disappointed everyone…( Participant 3)

#### Unmet expectations of motherhood

Many of the participants showed a desire to become mothers at one point in the future. They further mentioned that their aim for seeking help was to get a child at some point. One participant shared,

……..I have never got pregnant ever since I became of age and I do not know the experience of being pregnant and I long for this…. And my husband is longing for me to produce for him a child… I want to have a child I call my own… (Participant 12)

Some of the participants wished to have their own children especially after seeing other women with their own children and/or those who are pregnant.

… a friend may come to visit you and she is carrying her baby … then you to start thinking that I wish I also had a baby … hurting like that … seeing another woman with a baby then you wish that you also had a baby… such challenges a heartbreaking ….( Participant 1)

### Financial constraints

Almost all the participants mentioned having tried different treatment options, and some reported having failed to get treated due to the high costs that were involved. The issues that emerged out of this theme related to treatment options and financial constraints.

Most of the participants reported having faced lots of financial limitations during the process of treatment. Some clients reported not to have been able to carry out the requested investigations because of the cost attached to the investigations and could not even afford the prescribed medicines due to the cost. For example, one participant narrated:

……… the doctor said I needed to do an x-ray to check my tubes and it was not done at that particular hospital … so I went to another clinic and they told me the cost of the x-ray and it was expensive, and I couldn’t afford it and I had to give it time…… I took six months without going to the doctor and I have come back today and they have told me the same thing….( Participant 2)

Some participants reported that they were told that the only option of becoming a mother was to do IVF (in-vitro fertilization). Most respondents indicated that they lost all the hopes of being a mother at one point because of the high costs involved in the IVF process.

I was told I can try that treatment of IVF … I cannot afford it … and if I am to assess myself, I do not have anything I can sell to afford that treatment……I think it was not my calling to become a mother ….( Participant 3)

### Coping strategies of women living with involuntary childlessness

Following years of involuntary childlessness, women developed coping strategies to deal with involuntary childlessness; some women developed positive coping strategies while others copped negatively (Table 3).

**Table 3:** Coding tree for coping strategies of women living with involuntary childlessness

Theme	Categories	Codes
Positive coping strategies	Seeking treatment	I have visited traditional doctors, herbalists, and medical doctors/gynecologists, I have gone every where
	Spiritual coping	I put my trust in God
		I go to church, and it helps me cope
		I pray and read the bible
		I know God’s reward will be in heaven
		God created me for a different purpose
	Optimism	I reassure myself and I know everything will be fine
		What I do I love myself and I do not get stressed
		I am very hopeful I will get a child one day
	Acceptance	I Ignore negative comments because I can’t change my self
		I am patient with my self
		This is my fate and I take it as God’s will
	Family and social support	My family is Supportive, understanding, patient, my husband counsels me, this makes me feel better
		My partner provides, food, and money for treatment
		My friends are Supportive, they advise me without being judgmental
		I stay with my sister’s child who is like my own
Negative coping mechanism	Distractions	I concentrate on my work and make sure I accumulate money to keep my husband
		I ignore everyone and every comment
		I sometimes drink alcohol to forget everything
		I make sure I obey my husband and do everything he wants
	Social withdraw and isolation	I do not disclose my problems to any one as they may end up making me a topic of discussion
		I hide from people
		I avoid friends and anything that reminds me of my childlessness
		I avoid social gatherings
		When I cry, I feel better
	Negative self-concept	What did I do to God, if mad women are raped and become pregnant, then why not me who has a husband?
		I think my uterus was cursed
		I hate myself and I know I was cursed

### Positive coping strategies

Women developed positive coping strategies to involuntary childlessness to help them cope. The mental strength of self-belief and acceptance, seeking treatment, spiritual coping, and family social support were some of the coping strategies used by involuntary childless women in this study. These are each described below.

#### Seeking treatment

Because of involuntary childlessness, some of the women were able to cope with it by seeking out the available treatment options. Some traditional and modern medicine brought relief and gave them hope for a child in the future.

Most of the participants had used all treatment alternatives ranging from medical treatment, herbalists to consulting traditional doctors, and most of them could not even count the times they had consulted to solve their problem. This was manifest in the following quotes below:

… To be sincere they are so many places I have gone to, and I can’t count, (Participant 2)

She continued to narrate.

…… for the first time I went to herbalists but these drugs have no dose you take jerricans until you get tired, I got tired of them, then I started going to hospitals, today you go for a scan and you get different results from different places… however they have not told me of any problem that stops me from having a child and I have hope that I will get a child…(participant 2)

#### Spiritual coping

Some women sought solace in religious institutions, prayed to God, read the Bible, and accepted it as God’s will. This gave them hope to wait on God’s will while others believed that God created them for a different purpose and their reward is in heaven.

Some participants resorted to praying to God and hoping that one day God would answer their prayer of getting a child. Participants joined prayer groups, asked their churches to pray for them, and occasionally went to their respective prayer places. The participant believed that prayer encouraged them to keep hopeful and helped them cope with this situation,

…. mostly I put my trust in God…I usually go to Church and when I am from there my hopes of getting a baby are always high…they usually counsel us and by the end of it you have hope, and you also have faith that anytime God will grant your wish. (Participant 1)

#### Optimism

Some participants believed in themselves, continuously reassured themselves, practiced positive thinking, and concentrated on their work. The following examples are illustrative of self-belief.

…so what I do that I reassure myself …and tell myself positive words and keeping hoping. This gives strength to continue with my everyday work….… I taught myself that not having a child was not an end itself for someone to live…. so, I have learned to love myself, reassure myself …I console myself that maybe God did not want to me produce children…(Participant 10)

She continued to narrate:

…… I learned to be strong on my own…. I prayed, went to all pastors to pray for me, and somehow, I would come back home strong, and I knew that was God’s will…. (Participant 10)

#### Acceptance

Most participants mentioned that this situation is God’s will and they resorted to accepting the situation, paid no attention to people’s comments and this gave them the courage to move through each day.

Having no child is not the end of the world, I have come to accept it…. I concentrate on my Job… and I am happy…(Participant 5)

#### Family and social support

Lastly, women who received family and community support were able to cope with involuntary childlessness. The emotional support from the partner, family, and friends particularly helped the woman to cope with the apparent condition of not having children. This was ably backed by the expressions represented in the following quotes:

I have been able to bear this pain because my husband is supportive…. there is a time when I was desperate but he would tell me to be firm…that for him he has not been demanding from me to have a child…that when the situation is back to normal, he is very sure that I will be able to conceive… if it wasn’t for him I would have suffered so much...(Participant 9)

Some participants reported having been able to endure their situation because of close friends, workmates that kept on encouraging them and hanging out with them whenever they felt low.

Colleagues at work and my family have been supportive…. (Participant 11)

What I have done is to be sharing what I am going through with friends and getting counseling and comfort from my parents …. Close friends of mine have stood by me and have been a source of comfort… this has enabled me to stay sane and follow any advice they give me (Participant 5)

### Negative coping strategies

While some women were able to cope positively with involuntary childlessness, other women used negative coping strategies. This was described under the categories of distraction, social withdraw and isolation, and negative self-concept. The following extracts below show how women ineffectively coped with involuntary childlessness:

#### Distractions

Some participants reported finding distractions to help them forget their childlessness, like drinking alcohol, while others reported being so obedient to their husbands, concentrating on their work to make more money as a way of compensating for involuntary childlessness. This kept most of the women avoiding the actual problem and prevented them from accepting and hence negative coping.

….I concentrate on my job and I try to make as much money as possible because I have money more than my husband, he does not find reasons to send me away and besides, I help most of his family members…. (Participant 4)

I am very obedient to my husband…. I do everything that he wants… I am just still in this marriage because of his mercy so I have to be very obedient …..( Participant 5)

#### Social withdrawal and isolation

Other participants stated that whenever they felt so emotional, they would close themselves and cry in hiding; most of the participants avoided social gatherings and other places that reminded them of their childlessness.

…..the truth is I would close myself in a room and cry all day and night… as for my in-laws, I decided not to respond to their comments and ignored them… because nobody ever understood my pain….I never meet any of his family members just because of this reason (Participant 10)

There are times when I cry… and I can’t avoid it, because everything is just overwhelming …… usually when my mother-in-law abused me, I would cry… I keep on avoiding my in-laws and friends because they always ask me about children ….. Yet it’s not my wish not to have children (Participant 6)

#### Negative self-concept

Some participants perceived their bodies as though they were created with faults, while others blamed themselves for failing to have children.

…. It’s hard to go through this situation and I think it’s a curse …so you be there hurting all the time, asking God why this happening to me….. (Participant 6)

She continued to narrate,

…..I have never aborted any child and I do not use any family planning method… but some continuously abort but eventually give birth to babies… even there those who do not have men and are raped they get pregnant… whereas you who has a man who can look after you, you are unable to get pregnant (claps in anguish)… I know my body was cursed (Participant 6)

## Discussion

The study was conducted to explore the experiences and coping strategies of women living with involuntary childlessness in Uganda. Women, in this study were blamed, psychologically tortured, unsupported, and experienced feelings of distress and grief following failure to bear children. In addition, women had unstable marriages but also had financial constraints when seeking treatment; some women in this study coped positively while others had negative coping strategies.

In this study, women with involuntary childlessness were largely not supported by their partners, family, and society. Women were not allowed to own property even if their own income purchased it. Women were not involved in decision-making and felt that their identity as a woman in the family was taken away because they were not considered a wife. Their husbands and other family members were not supportive while seeking treatment. Women were left without any basic support, and they had to go through the whole experience as individuals [5, 15]. The extreme value placed on children in Uganda means that women are only recognized, valued, and accorded prestigious status through bearing children. Thus, failure to bear children translates to a lack of support and recognition in social settings[16].

In this study, most of the women experienced psychological trauma. The psychological torture was in the form of name-calling, blatant emotional abuse, internalized stigma, and blame. These results are consistent with those of the study in the Gambia that showed that when a marriage remains childless, some men abuse their wives verbally, emotionally, or even physically [17]. However, in the current study, no participant reported physical abuse. In other studies, women described how they are shouted at, cursed, and victimized by their families and the community at large [18]. Like in previous studies, women in this study were emotionally abused by the male partner’s family, who blamed the woman for failing to produce children [17, 19]. Similarly, women experienced self-inflicted stigma as they perceived themselves as a burden to the family. These experiences are comparable to those of Pakistan and American women. In both these countries, women felt stigmatized because of expectations of motherhood, for the case of married women [20].

The research findings indicated that women experienced negative feelings to a great extent, like sadness, anger, hopelessness, and disappointments, among others. However, some women indicated positive feelings like hope and faith. This is like a study done in Turkey where many childless women had negative feelings. Nonetheless, some women had positive feelings and hoped that one day God would answer their prayers [21, 22].

Another key finding is the desire for motherhood. The study results have indicated that all women were yearning to become mothers. This was voiced in their speech; some women had never even miscarried and wished to experience how it feels to become pregnant, go through labor, and raise their children. All these voices indicate the heaviness of grief that these women were going through. Women in Britain and Pakistan also expressed the same sentiments [20, 23]. This requires counselors and nurses on the team working on involuntarily childless women to provide psychological and emotional support to enable these women to go through this experience without affecting their daily living, more so, they should be provided with a calm setting within the hospital where they can meet and share experiences and covert their negative experiences into positive experiences. In addition, there is need for the government to equip all higher level facilities with necessary equipment and healthcare providers to provide treatment to women living with involuntary childlessness at a subsided cost to reduce the burden of childlessness.

Some women perceived that their husbands had found other women in search of a child, while other men were thought to be involved in intimate relations outside their marriage without fear of being seen by their wives. As such, some women were in fear that their husbands might end up getting other women [24]. However, a related study showed that involuntarily childless women were also more likely to engage in marital infidelity than women with children [25]. Another study done in Pakistan had similar results indicating that women had insecurities specifically the fears of marital breakdown, based on the worry that their husband would leave them to find a new wife capable of bearing children. Such men were concerned about patrilineal inheritance [15]

As a result of childlessness, other participants reported that they were sent away from their marital homes by their in-laws, and on other occasions, their in-laws suggested other women to their husbands. In this study, one of the participants had suffered a divorce. As expected, much frustration came in. This was similar in several studies where women expressed thoughts of suicide as aggravated by instability within their relationships [17, 22]. On the contrary, a study done in Indonesia indicated that women were supported by their in-laws and were never questioned about children at all [18]. The involvement of social workers and community leaders can help in advocating for these women’s rights.

In this study, all women believed in the culture in which motherhood is the only way to be considered feminine, Women felt that they had failed to meet their expectations as everyone expected them to bear children soon after their marriage. Certainly, this caused self-blame, and they felt guilty and disappointed for having failed to have children. The study carried out in Zimbabwe among childless women revealed that women were guilty of having no children, they avoided social gatherings and were referred to as bewitched women, and they felt like they have failed their community [11]. However, this was contrary to the study done in UK, where women in reproductive age, and were fertile but did not want to have children, as some women did not want the responsibilities that comes with motherhood [26].

Women in this study saw motherhood as an inevitable part of becoming a fulfilled woman. This was complicated by the pressure from spouses and relatives, who kept on demanding a child as their failure to have children was seen as selfishness. This is similar to several other studies where women lost their identity as women due to failing to bear children, they kept on yearning to have children to regard themselves as fulfilled women and stop being judged as mean, child-hating, and materialistic [27]. Continuous counseling and the creation of support groups can help these women share their experiences and find solutions.

In this study, some women did not take the prescribed medicines or finish the recommended dose, while others did not even carry out the recommended investigations due to the cost attached to each. Some women wished to do In-vitro fertilization but could not afford it. Other studies have also revealed that women found hardships in paying treatment expenses and requested the government to reduce costs for involuntary childless treatment in private sectors [21]. In another related study done in Gambia, the participants failed to carry out any test and get any form of treatment due to the cost attached [7, 28].

These findings were like those of several studies where women did not access the necessary treatment due to financial constraints. Some women needed to undergo assisted reproductive technology treatments, and this was not achieved due to the too much cost attached to it [22, 28]. The government should support women living with involuntary childlessness to have some investigations and treatment done at the government facilities at a lesser cost.

The study had some limitations, the nature of the study topic made some women more emotional, this could have hindered open expression and information sharing about their experiences during the interview and women were mandated to remember some of the experiences that occurred in the past, remembering or not remembering experiences or events that occurred at some point in the past could have affected the nature of the experience. In addition, the study was conducted in one hospital with urban residence, further studies are recommended to address the rural urban residence and its relationship with the social pressure, family support and difference in wealth index.  

### Coping with involuntary childlessness

In this study, coping strategies are specific efforts, both behavioral and psychological, that women employ to master, tolerate, reduce, or minimize stressful events of involuntary childlessness [29]. Women with involuntary childlessness acknowledged emotional, social, physical, and financial challenges associated with involuntary childlessness, and they adopted coping strategies to move beyond the effects of involuntary childlessness. However, some adopted positive strategies while the majority adopted negative ones.

In the present study, women reportedly used different treatment options; namely, they visited traditional doctors, witch doctors, and consulted medical doctors. Most of the women had used herbal medicines in conjunction with standard medicines. Women used every treatment they were told about in search of a child. Similarly, women in Mali sought all forms of treatment, ranging from traditional to medical treatment [30]. Also, women visited hospitals and herbalists several times. Others could not even count the number of times and used all treatment remedies given. This was consistent with findings from a study among Turkish women where involuntarily childless women searched complementary medicine as an alternative to medical treatment. Women had also visited several places in search of the treatment of involuntary childlessness [21].

Other participants reassured themselves, kept only positive comments, ignored negative comments and thoughts, and being hopeful helped them to cope. The women found no reason to express their pain to the public; women commented that no one could understand the pain of involuntary childlessness, especially those with children, and they decided to keep it within their hearts and not to disclose their pain. This finding is similar to findings of a study from Mali where; women believed in self-isolation as a way of coping, the pain was not disclosed to anyone as they believed nobody understood them [30].

It is also important to note that there were participants who reported coping by crying in hiding, especially after feeling so much tension. This was similar to the findings of a study in Gambia where women with involuntary childlessness coped by crying in hiding; others cried within their hearts to overcome the bitterness of childlessness [30]. Moreover, women in this study avoided places that reminded them of their childlessness; they avoided social gatherings that have children and places with pregnant women.

In this study, family support was significant in helping some women cope. However, most women did not receive support from their families. Women supported by their husbands and other family members coped positively with involuntary childlessness, which helped them greatly. In Vadodara, families and other social networks played an important role in helping childless women cope with the feeling of guilt, worthlessness, depression, and low self-esteem, which they often experienced [31, 32]. Other studies show that involuntarily childless women find their spouse, family, and relatives to be a source of support [23], although most women in this study did not receive support from their family and relatives.

## Conclusion

From the study findings, involuntary childlessness is a public health concern although it has received very little to no attention from policymakers in Uganda. Women with involuntary childlessness face several negative consequences even when the problem stems from their partners, women face stigma from within their families and their communities in which they live. They reported to be having challenges with their relationships, they receive hurtful comments and this affects their emotional wellbeing. Women living with involuntary childlessness were excluded from social gatherings and some other women excluded themselves while trying to cope with involuntary childlessness.

Women living with involuntary childlessness lacked social support and financial support, this affected their choices of treatment as many treatment options and investigations were not available in the government facilities. This demands a need to prioritize and incorporate the treatment of involuntary childlessness into existing reproductive health services to reduce the burden of childlessness. There is a need to provide empathetic care and counsel, providing mechanisms for coping like group therapy. Advocacy for subsided treatment and investigations are needed to aid these women in accessing the treatment.

## Data Availability

The datasets used and/or analyzed during the current study are available from the corresponding author on reasonable request.

## Abbreviations

AHSPR: Annual health sector performance report
ICF: International classification of functioning, disability, and health
IVF: Invitro fertilization
KNRH: Kawempe National Referral Hospital
MoH: Ministry of health
NDS: National demographic survey
UBOS: Uganda Bureau of statistics
WHO: World Health Organization
